# Kinetic Studies that Evaluate the Solvolytic Mechanisms of Allyl and Vinyl Chloroformate Esters

**DOI:** 10.3390/ijms14047286

**Published:** 2013-04-02

**Authors:** Malcolm J. D’Souza, Aaron F. Givens, Peter A. Lorchak, Abigail E. Greenwood, Stacey L. Gottschall, Shannon E. Carter, Dennis N. Kevill

**Affiliations:** 1 Department of Chemistry, Wesley College, 120 N. State Street, Dover, DE 19901-3875, USA; 2 Department of Chemistry and Biochemistry, Northern Illinois University, DeKalb, IL 60115-2862, USA

**Keywords:** allyl chloroformate, vinyl chloroformate, mechanisms, solvolysis, nucleophilicity, ionizing power, π electron cloud, Grunwald-Winstein equation, Linear Free Energy Relationships (LFERs)

## Abstract

At 25.0 °C the specific rates of solvolysis for allyl and vinyl chloroformates have been determined in a wide mix of pure and aqueous organic mixtures. In all the solvents studied, vinyl chloroformate was found to react significantly faster than allyl chloroformate. Multiple correlation analyses of these rates are completed using the extended (two-term) Grunwald-Winstein equation with incorporation of literature values for solvent nucleophilicity (*N*_T_) and solvent ionizing power (*Y*_Cl_). Both substrates were found to solvolyze by similar dual bimolecular carbonyl-addition and unimolecular ionization channels, each heavily dependent upon the solvents nucleophilicity and ionizing ability.

## 1. Introduction

Profound interest in the use of choroformate esters began during World War I due to their ability to cause immense physiological damage [1–3]. Beginning in the 1940s interest in more advantageous agricultural applications commenced with the use of chloroformate esters in a number of herbicide, fungicide, and insecticide formulations [4]. During the past seventy years, both saturated and unsaturated chloroformate esters have gained greater importance commercially due to their many applications as protecting groups and as precursors in the synthesis of novel prodrugs [5–7].

Quantitative linear free energy relationships (LFERs) such as the simple and extended Grunwald-Winstein [Equations (1) and (2)] [8] have been successfully employed [9,10] to elucidate the varying solvolytic mechanism of reaction of such chloroformate esters in a variety of hydroxylic solvents with widely varying nucleophilic and ionizing abilities.

In Equations (1) and (2), *k* is the specific rate of solvolysis of a chloroformate ester in a given solvent and *k_o_* is the specific rate of that same substrate in 80% ethanol (taken as the standard solvent), *m* represents the sensitivity to changes in the solvent ionizing power *Y*_X_ (based on the solvolysis of 1- or 2-adamantyl derivatives) [11–16], *c* is a constant (residual) term, and *l* is the sensitivity to changes in solvent nucleophilicity *N*_T_ (determined from the rates obtained for the solvolysis of the *S*-methyldibenzothiophenium ion) [17,18].

(1) $$\text{log }(k/k_{o})=mY_{\text{X}}+c$$

(2) $$\text{log }(k/k_{o})=lN_{\text{T}}+mY_{\text{X}}+c$$

Solvolytic data for a variety of substituted phenyl chloroformates have been documented and thorough Grunwald-Winstein (G-W) analyses of the available experimental results using Equation (2) have been published [9,10,19–31]. The kinetic solvent isotope effects (KSIEs) in methanol and methanol-d (*k*_MeOH_/*k*_MeOD_) for the substituted phenyl chloroformates indicate that general-base catalysis is operating, and its importance decreases with an increase in electron-donating ability of the substituent [21,26,30,32,33]. For the parent phenyl chloroformate (**1**), over a full-range of 49 pure and binary solvents of widely varying nucleophilicity and ionizing power values, the G-W [Equation (2)] analyses resulted in an *l* value of 1.66 and an *m* value of 0.56 [27]. It was suggested [10,23,27,30,31] that such *l* (bond making) and *m* (bond breaking) values be taken as typical values that are to be expected for substrates that proceed with a rate-determining addition step in a stepwise carbonyl addition-elimination process. Other research groups also proposed homogenous stepwise mechanisms for **1**, where the formation of a zwitterionic tetrahedral intermediate is the rate-determining step [34–38]. Like **1**, recent G-W analyses for the solvolysis of 1- and 2-naphthyl chloroformates also show that the bimolecular addition-elimination pathway is robust in a wide variety of solvents [39].

For primary alkyl chloroformates the rate-determining carbonyl-addition process is the dominant mechanism in a majority of the solvents studied, and in the fluoroalcohols (solvents with low nuclophilicity and high ionizing ability), an ionization mechanism with strong nucleophilic solvation of the developing acylium ion is favored [40–45]. The strongest initial evidence for such a rate-determining addition-elimination process came from a study that compared the experimental rates obtained for *n*-octyl chloroformate and *n*-octyl fluoroformate in identical solvents [42] (of widely varying nucleophilicity and ionizing power values). The comparison showed a F:Cl rate ratio that was greater than unity. This ratio is consistent with the addition step of carbonyl-addition process being rate determining. Multiple studies contrasting other alkyl haloformate esters [46–50] show that the fluoroformates that have a stronger carbon-fluorine bond solvolyze faster than their corresponding chloroformate esters and such a high F:Cl ratio also endures in benzoyl halides [33,51,52].

On the other hand for secondary chloroformates, an evaluation of the kinetic rate data obtained for the bulk of the solutions studied resulted in a proposal that isopropyl chloroformate [53] and 2-adamantyl chloroformate [54] show dominant solvolysis-decomposition with loss of the CO_2_ molecule. This mechanism parallels the behavior observed for tertiary 1-adamantyl chloroformate in all of the solvents studied where the major products are the decomposition product, 1-adamantyl chloride, and an ether and/or the alcohol (depending on the solvent components) [55].

Competing addition-elimination and solvolysis-decomposition patterns were observed for benzyl and *p*-nitrobenzyl chloroformates [56]. The dominance of one pathway over the other was shown to be strongly dependent on the solvent’s nucleophilicity value and its ionizing power ability [56].

Isopropenyl chloroformate (**2**) was the first alkenyl chloroformate to be exhaustively analyzed using Equation (2) in 51 solvents [57]. A bimolecular tetrahedral addition-elimination pathway was the predominant reaction mechanism observed, and a superimposed ionization pathway was shown to make a significant contribution in the four 97%–70% 1,1,1,3,3,3-hexafluoro-2-propanol (HFIP) and 97% 2,2,2-trifluoroethanol (TFE) mixtures with water.

So far the two alkynyl chloroformates to be evaluated using the extended Grunwald-Winstein Equation [Equation (2)] are; propargyl [58] and 2-butyn-1-yl chloroformate [31]. For propargyl chloroformate, the tetrahedral stepwise addition-elimination pathway was confirmed for all 22 solvents studied [58]. 2-butyn-1-yl chloroformate differs from the propargyl substrate by the presence of an adjoining methyl group on the β-triple bond. For this alkynyl containing compound, the addition-elimination pathway again dominates the spectrum of the solvents considered, but the reaction switches over to an ionization channel in the two most strongly hydrogen-bonding solvents studied, 97% and 90% HFIP [31].

The alkenyl chloroformates, allyl (**3**) and vinyl (**4**) chloroformates have gained recent media attention due to their meaningful use in surface coating technology to create commercial bacterial-resistant amphiphilic polymers [59]. The solvolyses of allyl chloroformate (**3**) was recently evaluated in 35 solvents at 35.0 °C [60]. The authors Koh and Kang proposed a lose bimolecular S_N_2 reaction based on the magnitudes of the Grunwald-Winstein *l* and *m* values obtained, the activation parameters and KSIE determinations, and the product selectivity data in alcohol/water mixtures [60]. The rate data reported for the ethanolysis of allyl chloroformate at 25.0 °C [60] was found to be smaller than the corresponding value obtained for benzyl chloroformate (**5**) at the same temperature [56].

In general, studies have shown that conjugated allylic substrates show enhanced reactivity due to the proximity of the *pi* system of the carbon-carbon double bond [29,33,61]. Additionally due to the possibility of increased resonance stabilization in the cation, allyl, benzyl, and benzoyl substrates typically tend to favor stepwise unimolecular S_N_1 (dissociative) reactions [29,33,51,52,62–65]. An allyl cation with two resonance contributors is shown to have approximately the same stability as a secondary alkyl cation [66]. On the other hand reactivity at an sp^2^ carbon of vinyl substrates is dependent on whether the substrate is activated or unactivated, for alkyl-substituted vinyl triflates are shown to solvolyze through ion-pair mechanisms where nucleophilic solvation is important in substrates where the β hydrogen is *trans* to the leaving group [66–73].

**Figure 1:**
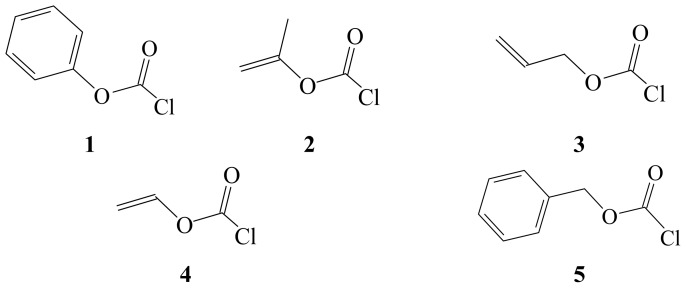
Molecular structures of phenyl chloroformate (**1**), isopropenyl chloroformate (**2**), allyl chloroformate (**3**), vinyl chloroformate (**4**) and benzyl chloroformate (**5**).

In Figure 1, we present the three-dimensional arrangements of 1–5. The molecular structures drawn clearly show the proximity of the π-bond to the ether oxygen. This environment could influence the polarization of the bonding to the ether oxygen and as a consequence, this would affect any inductive and/or mesomeric effects observed. To further probe the potential of any accelerating or deshielding π-conjugation effects, we now present the analysis of our experimental first-order specific rates of solvolysis of allyl (3) and vinyl (4) chloroformates at 25.0 °C. We also reanalyze the Koh and Kang data [60] for 3 at 35.0 °C. We compare the solvolytic data obtained at 25.0 °C to the available literature values of phenyl (1) [21–23,26,27] and benzyl (5) [56] chloroformate at the same temperature.

## 2. Results and Discussion

The rates of solvolysis for allyl (3) and vinyl (4) chloroformate are measured in 20 and 17 pure and binary aqueous organic solvents respectively. These rates determined at 25.0 °C are listed in Table 1, and the solvents include mixtures of aqueous fluoroalcohols.

For allyl chloroformate (3), there is a gradual increment in the specific rate of solvolysis with the increase in water content in mixtures with ethanol, methanol, acetone, and TFE. In the aqueous HFIP mixtures, the rate of 3 is the greatest in the very strongly hydrogen bonding 97% HFIP. The 80% EtOH and 100% MeOH values documented in Table 1 for 3 are within the margin of error when compared to those reported at 25.0 °C by Koh and Kang [60]. However our ethanolyses rate for 3 is almost three times faster than the previously reported value [60]. We have since repeated this determination six times using different batches of purified solvent to make sure that our reported EtOH value is correct.

The rates of solvolysis for vinyl chloroformate (4) also rise as the water content of the binary aqueous mixtures increase. In the TFE-EtOH mixtures, the rise in the specific rates intensifies with the increase in ethanol content. This illustrates the importance of the role of solvent nucleophilicity at the developing transition state for solvolyses in these mixtures.

In Table 1, we also list the 25.0 °C values for phenyl (1) [21–23,26,27] and benzyl (5) [56] chloroformate that are obtained from the literature for the common solvents that 3 and 4 were studied in. In the TFE-EtOH mixtures and in the aqueous EtOH, MeOH, and acetone solvents, a general trend of *k*_4_ >> *k*_1_ > *k*_5_ ≈ *k*_3_ is observed. In these particular solvents, 1 and 5 have been shown to solvolyze by a bimolecular addition-elimination process via the formation of a tetrahedral intermediate [22,23,27,56].

For **3**, in 30 solvents examined (excluding the TFE-EtOH mixtures), Koh and Kang claimed [60] that a predominant bimolecular S_N_2 type mechanism was occurring since the G-W analysis of their rates for **3** [at 35.0 °C and using Equation (2)] resulted in an *l* value of 0.93, an *m* value of 0.41, and *R* = 0.964. In Table 2, we have reported the results that we obtained for substrates **3**, and **4** after a multiple correlation analyses employing Equation (2).

A reanalysis using Equation (2) of the published data for **3** in all 35 solvents studied at 35.0 °C resulted in an *l* value of 0.98 ± 0.06, an *m* value of 0.44 ± 0.03, *c* = −0.04 ± 0.05, *R* = 0.945, and a *F*-test value of 132 (reported in Table 2). These values are essentially the same as those reported by Koh and Kang for 30 solvents [60] and this statistical outcome demonstrates that in this particular case there is little gained by for excluding the TFE-EtOH solvents in such a G-W analysis.

In the highly ionizing aqueous HFIP and aqueous TFE binary mixtures a nucleophilic addition-elimination mechanism was still shown to dominate in **1**[22,23,27], while a solvolysis-decomposition type process after formation of a cationic transition state was proposed for **5**[56]. On the other hand for the alkenyl containing isopropenyl chloroformate (**2**), it was proposed [57] that a superimposed unimolecular (S_N_1) type ionization process was making a significant contribution in 97%–70% HFIP, and 97% TFE due to the formation of a resonance stabilized intermediate carbocation.

**Figure 2:**
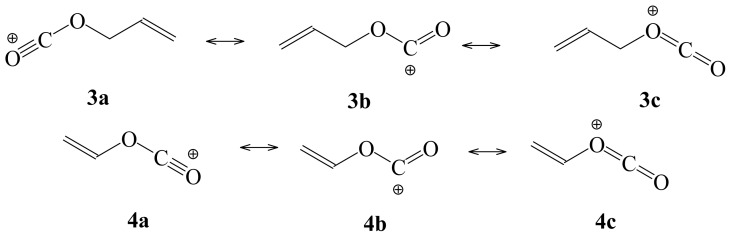
Possible carbonyl cation resonance structures for compounds **3** and **4**.

In Figure 2 (above), we show the important resonance contributors for the two alkenoxy carbonyl cations considered in this study. These delocalized structures attest to the immense possibility that like **2**, **3** and **4** could show significant unimolecular S_N_1 character in the highly ionizing aqueous fluoroalcohol mixtures.

For **3** at 35.0 °C a G-W analysis obtained on the exclusion of the data points [60] in the TFE (aq) and HFIP (aq) solutions resulted in, *l* = 1.43 ± 0.13, *m* = 0.52 ± 0.03, *c* = 0.10 ± 0.06, *R* = 0.954, and *F* = 127. These *l* and *m* values are within the range of those obtained for **1**, strongly suggesting that a carbonyl addition would be the favored process in the remaining 28 solvents analyzed. In just the seven TFE (aq) and HFIP (aq) mixtures, we obtain 0.93 ± 0.12, 0.66 ± 0.14, −0.84 ± 0.30, 0.974, and 36 for *l*, *m*, *c*, *R*, and *F* respectively. This *l*/*m* ratio of 1.41 obtained for the TFE and HFIP mixtures is similar in magnitude to that previously observed for the solvolyses of acetyl chloride (*l*/*m* ratio of 1.19) [74] and 4-morpholinecarbonyl [75] chloride (*l*/*m* ratio of 1.12). Such ranges in *l*/*m* ratios were reported to articulate unimolecular ionization (S_N_1) pathways with considerable nucleophilic solvation at the developing carbocation [74,75].

In this study, we document the rates obtained for allyl chloroformate (**3**) at 25.0 °C (Table 1). A G-W analysis (Table 2) for 12 solvents excluding the aqueous fluoroalcohol mixtures (TFE and HFIP), show *l* = 1.46 ± 0.19, *m* = 0.37 ± 0.09, *c* = 0.10 ± 0.08, *R* = 0.943, and *F* = 37. In order to identify the exact ionizing solvents (with high fluoroalcohol content) where the mechanisms displayed the greatest overlapping traits, we then carried out a G-W analysis in a different set of 12 solvents which eliminated the data points in 80T–20E, 97%–70% TFE (aq) and 97%–70% HFIP (aq). We obtained 1.78 ± 0.18, 0.43 ± 0.07, 0.14 ± 0.06, 0.965, and 61, for *l*, *m*, *c*, *R*, and *F* respectively (Table 2). For these 12 solvents, the much improved *R* and *F*-test values advocate that these particular *l* (1.78) and *m* (0.43) values obtained are the deciding contributions that indicate the amounts of bond making and bond breaking at the tetrahedral transition state for **3**.

**Figure 3:**
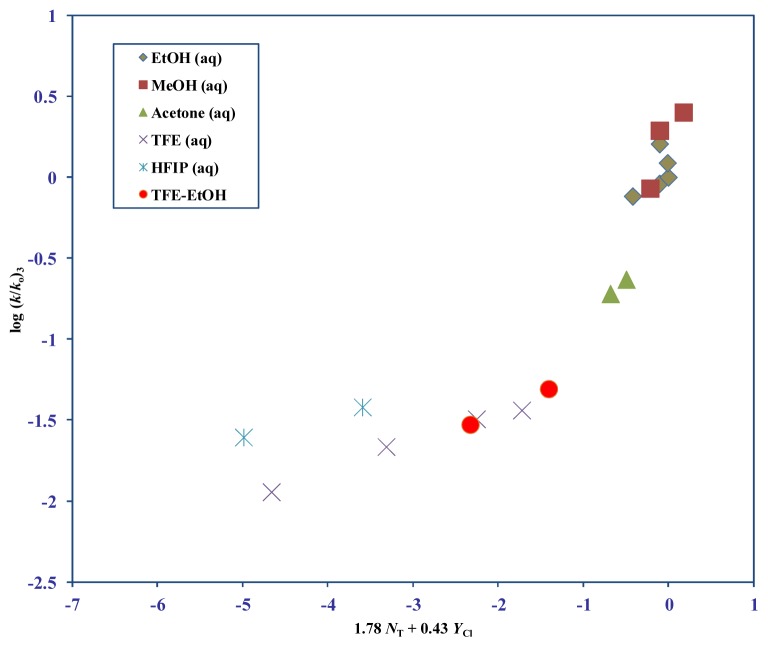
The plot of log (*k*/*k*_o_) for 3 at 25.0 °C against 1.78 *N*_T_ + 0.43 *Y*_Cl_.

In Figure 3, a plot of log (*k*/*k*_o_) for 3 at 25.0 °C against 1.78 *N*_T_ + 0.43 *Y*_Cl_ is shown. The seven fluoroalcohol containing solvents (97%–90% HFIP, 97%–70% TFE, and 80T–20E) are excluded from the G-W calculation but are added to the plot to show the extent of their deviation from the line-of-best-fit.

**Figure 4:**
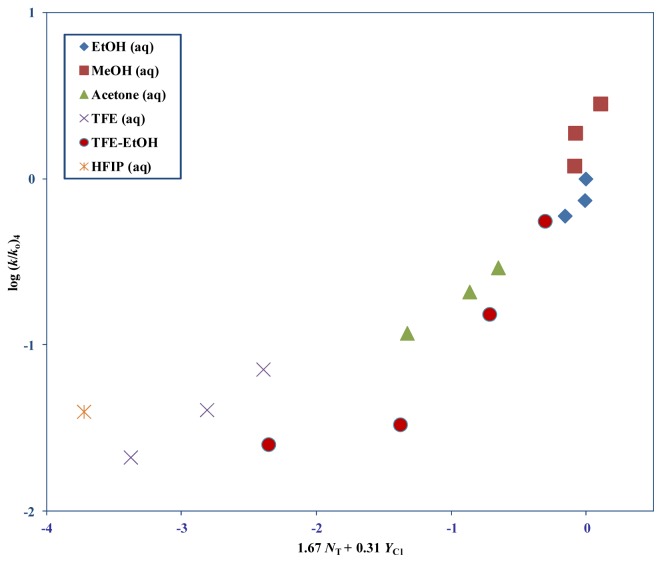
The plot of log (*k*/*k*_o_) for 4 at 25.0 °C against 1.67 *N*_T_ + 0.31 *Y*_Cl_.

A G-W analysis of 4 in the 12 more nucleophilic solvents (excluding, 80T–20E, 70% HFIP, and 90%–70% TFE) resulted in, *l* = 1.67 ± 0.19, *m* = 0.31 ± 0.07, *c* = 0.10 ± 0.09, *R* = 0.941, and *F* = 35 (Table 2). In Figure 4, a plot of log (*k*/*k*_o_) for 4 at 25.0 °C against 1.67 *N*_T_ + 0.31 *Y*_Cl_ is shown. The five excluded fluoroalcohol solvents were not included in the multiple regression analyses but were added on to demonstrate the intensity of the divergence from the regression line.

The *l* and *m* values obtained for **4** are numerically analogous to those calculated for **3**. Such *l* and *m* values are consistent with the proposed bimolecular pathway through a tetrahedral intermediate formed by the rate determining addition of the solvent at the carbonyl carbon. The slightly reduced *m* values for **3** and **4** could imply a decreased need for solvation of the developing negative charge on the carbonyl oxygen in the sp^3^ hybridized tetrahedral transition state.

For **4**, in the five remaining fluoroalcohol mixtures (80T–20E, 70% HFIP, and 90%–70% TFE) we get 0.80 ± 0.03, 0.59± 0.01, (−1.31) ± 0.03, 0.999, and 578 for *l*, *m*, *c*, *R*, and *F* respectively. This *l*/*m* ratio is 1.36. For **3** and **4** the amount of dispersion (Figures 3 and 4) observed in these fluoroalcohols and the very similar fractional contributions towards the sensitivities of solvent nuclophilicity (*l*) and solvent ionizing power (*m*), suggests the definitive probability of virtually identical reaction channels occurring.

The ethanolyses rate of isopropenyl chloroformate (2) at 25.0 °C was reported to be (110 ± 6) × 10^−5^[57]. A comparison of this pure EtOH data point together with the other values listed in Table 1 show, *k*_4_ > *k*_2_ >> *k*_1_ > *k*_5_ ≈ *k*_3_. Therefore in the pure alcohol and aqueous EtOH, MeOH, and acetone solvents the increases in of the reaction rates observed with increasing solvent nucleophilicity can be attributed to the inductive ability of the different R groups in 1–5. Our observations based on the progression of the magnitude of these rates firmly indicates that the vinyl group in 4 (in these particular solvents) is very electron withdrawing via induction, and this especially holds true when its effect is compared to the responses observed due to the phenyl (1), benzyl (5), and allyl (3) groups. Consistent with this view vinyl has a polar substituent constant (σ^+^) of 0.653.

For **3** in deuterated MeOH, a *k*_MeOH_/*k*_MeOD_ value of 2.16 was observed at 35.0 °C [60]. The large KSIE value observed for 3, together with the large *l* parameters in the more nucleophilic solvents calculated for **3** and **4**, also indicate the strong possibility of general-base catalysis [21,26,30,32,33] to the nucleophilic attack in both **3** and **4**.

For **2** in 97% HFIP and 97% TFE at 25.0 °C, values of 3.23 × 10^−7^ and 5.94 × 10^−7^ are computed from the rates that we reported at different temperatures [57] and the Arrhenius equation. An examination of these calculated rates for 2 together with the rates for 1, 3, and 5 (listed in Table 1) in the highly ionizing and very strongly hydrogen bonding 97% HFIP (*Y*_Cl_ value of 5.17), we get *k*_5_ > *k*_3_ > *k*_2_ >> *k*_1_. In comparison, in the moderately enhanced hydrogen bonding but less ionizing 97% TFE (*Y*_Cl_ value of 2.83), the rate trend is *k*_5_ > *k*_3_ > *k*_2_ ≈ *k*_1_ (no rate value is available for 4 in 97% HFIP and 97% TFE). Even in 97% HFIP and 97% TFE, phenyl chloroformate (1) favors an addition-elimination pathway [23,27] so it is not surprising that its rate in these two highly ionizing solvents is the slowest.

**Figure 5:**
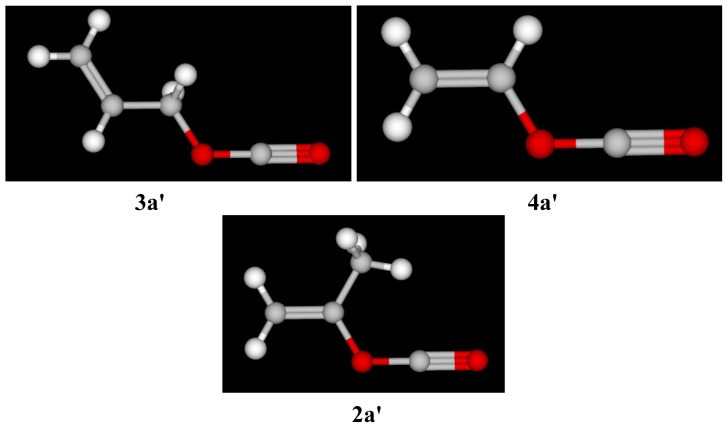
The 3-D images for the resonance conformers for the carbonyl cations of allyl chloroformate (3a'), vinyl chloroformate (4a'), and isopropenyl chloroformate (2a') are shown.

The 3-D images shown in Figure 5 are for a single resonance contributor formed within the developing acylium ion in 2–4. Our experimental evidence now suggests that such ionic species would be expected for 2–4 in the highly ionizing fluoroalcohols. A closer inspection reveals that in 3a', the delocalized π electron cloud of the alkenyl bond is far apart (and out of the plane) for any appreciable orbital overlap. However our investigation of the rate trends in 97% HFIP and 97% TFE show that the π systems that are further away from the reaction center are indeed getting involved through positive hyperconjugation in the stabilization the cationic center. This rate sequence is consistent with the superiority in resonance stabilization that is to be expected within the benzylic carbocation since in these two solvents, 5 is said to favor a solvent-decomposition process with a loss of a CO_2_ molecule [56].

In 70% HFIP, the only common fluoroalcohol-water mixture that 1–5 are studied in at 25.0 °C, a rate value of (2.54 ± 0.09) × 10^−5^ is listed for 2 [57], and the rate trend is *k*_4_ > *k*_5_ ≈ *k*_1_ > *k*_2_ > *k*_3_. In 90% TFE, we observe *k*_4_ > *k*_5_ > *k*_1_ > *k*_3_. Furthermore as solvent nucleophilicity increases with higher water content in going to 70% TFE, the rate trend moves towards *k*_4_ > *k*_1_ > *k*_5_ > *k*_3_ and in 50% TFE, the rate order remains as *k*_1_ > *k*_5_ > *k*_3_.

From these rate orders we can theorize that for the three alkenyl chloroformate ester solvolyses, the presence of dual side-by-side mechanisms is in response to pronounced competing mesomeric and inductive effects.

## 3. Experimental Section

The allyl chloroformate (97%) and the vinyl chloroformate (99%) were obtained from the Sigma-Aldrich Chemical Company and were used as received. Solvents were dried and purified as described previously [23]. A substrate concentration of approximately 0.005 M in a variety of solvents was employed. The rate constants for the slow solvolytic reactions were determined by the trimetric method [23] and the faster rates were followed by conductance, at appropriate time intervals [74]. For some of the runs, calculation of the specific rates of solvolysis (first-order rate coefficients) was carried out by a process in which the conventional Guggenheim treatment [76] was modified [77] so as to give an estimate of the infinity titer, which was then used to calculate for each run a series of integrated rate coefficients. The specific rates and associated standard deviations, as presented in Table 1, are obtained by averaging all of the values from, at least, duplicate runs.

Multiple regression analyses were carried out using standard Microsoft statistical packages (Excel 2010; Microsoft Corporation: Redmond, WA, USA, 2010) and calculations for the Guggenheim treatments were performed on commercially available software (SigmaPlot, version 9.0; SYSTAT Software Inc.: San Jose, CA, USA, 2005). The 3-D images presented in Figure 4, were computed using the KnowItAll^®^ Informatics System (ADME/Tox 2008; BioRad Laboratories: Philadelphia, PA, USA, 2008).

## 4. Conclusions

For the alkenyl chloroformate esters dual mesomeric and inductive effects are found to be in constant competition and their impact dominates the solvolyses of isopropenyl (**2**), allyl (**3**), and vinyl (**4**) chloroformate. In **4** as a result of the proximity of the π-electrons of the carbon-carbon double bond to the reaction center, its rates of reaction are very much faster when compared to those of **3** in all of the solvents studied.

For **3** and **4** G-W analyses using the two-term Equation (2) results in *l* and *m* sensitivities that forecast concurrent bimolecular tetrahedral carbonyl-addition and unimolecular ionization mechanisms. Such solvent-dependent synchronous mechanisms were also previously proposed for **2**[57]. For the alkenyl chloroformates **2**–**4**, in pure EtOH (which is very nucleophilic and low ionizing) the addition-elimination mechanism dominates, and at the other extreme in 70% HFIP, the ionization mechanism was found to be in control.

The molecular structure of **2** differs from that of **4** because of the presence of a methyl group on the α-carbon of **4**. The hyperconjugative (electron releasing) ability of this methyl group has a considerable effect on the reaction rates, and hence **2** is slower than **4** in EtOH and 70% HFIP. On the other hand, **2** is faster than **3** in EtOH and 70% HFIP, but slower in 97% HFIP and 97% TFE. In the latter fluoroalcohol mixtures, the additional presence of a methyl group (shown in **2a'**) cannot efficiently stabilize the developing cationic S_N_1 transition state and hence, **3** exhibits an accelerated rate in 97% HFIP and 90% TFE (when compared to **2**).

## Figures and Tables

**Table 1:** Specific rates of solvolysis (*k*) of 3 and 4 in several binary solvents at 25.0 °C indicated by (T) when determined by titration, otherwise determined by conductivity measurements, and literature values for 1, 5, *N_T_and Y_Cl_*.

Solvent (%) a	1 b 10^5^*k*, s^−1^	3 (T) 10^5^*k*, s^−1^ c	4 10^5^*k*, s^−1^ c	5 d 10^5^*k*, s^−1^	*N_T_*^e^	*Y_Cl_*^f^
100% EtOH	260	11.1 ± 0.16	742 ± 1	5.16	0.37	−2.50
90% EtOH	389	13.4 ± 0.7	921 ± 3	12.9	0.16	−0.90
80% EtOH	503	14.7 ± 0.4	1252 ± 19	17.7	0.00	0.00
70% EtOH	546	18.0 ± 0.6		21.5	−0.20	0.78
60% EtOH	658	23.4 ± 0.9		25.6	−0.38	1.38
100% MeOH	695	12.5 ± 0.7	1485 ± 13	18.8	0.17	−1.20
90% MeOH	1290	28.4 ± 1.5	2331 ± 42	38.4	−0.01	−0.20
80% MeOH	1670	36.7 ± 2.0	3500 ± 55	55.4	−0.06	0.67
60% MeOH	2220	38.5 ± 0.8			−0.54	2.07
90% Acetone	23.8		146 ± 2		−0.35	−2.39
80% Acetone	68.8		260 ± 4	2.13	−0.37	−0.80
70% Acetone	125	2.79 ± 0.14	365 ± 3	4.23	−0.42	0.17
60% Acetone	195	3.41 ± 0.14		7.62	−0.52	1.00
97% TFE (*w*/*w*)	0.0570	0.166 ± 0.016		1.93	−3.30	2.83
90% TFE (*w*/*w*)	1.15	0.317 ± 0.017	26.2 ± 0.3 (T)	2.37	−2.55	2.85
80% TFE (*w*/*w*)	7.02		50.5 ± 1.2	3.44	−2.19	2.90
70% TFE (*w*/*w*)	17.4	0.467 ± 0.021	88.6 ± 0.9	4.82	−1.98	2.96
50% TFE (*w*/*w*)	63.5	0.531 ± 0.022		9.39	−1.73	3.16
80T-20E	2.43	0.434 ± 0.016	31.6 ± 0.1 (T)	0.692	−1.76	1.89
60T-40E	17.5	0.723 ± 0.050	43.2 ± 0.1 (T)	0.993	−0.94	0.63
40T-60E	57.7		178 ± 1	2.19	−0.34	−0.48
20T-80E	169		696 ± 1	3.90	0.08	−1.42
97% HFIP (*w*/*w*)	14.8 × 10^−4^	1.80 ± 0.12		13.8	−5.26	5.17
90% HFIP (*w*/*w*)	0.166	0.362 ± 0.023		11.5	−3.84	4.41
70% HFIP (*w*/*w*)	10.5	0.553 ± 0.021	49.5 ± 0.2	11.3	−2.94	3.83

a Substrate concentration of *ca.* 0.0052 M; binary solvents on a volume-volume basis at 25.0 °C, except for TFE-H_2_O and HFIP-H_2_O solvents which are on a weight-weight basis. T-E are TFE-ethanol mixtures;

b References [22,23,27];

c With associated standard deviation;

d Reference [56];

e References [17,18];

f References [11–16].

**Table 2:** Correlation of the specific rates of reaction of **3**, and **4**, using Equation (2).

Substrate	*n*^a^	*l*^b^	*m*^b^	*c*^c^	*R*^d^	*F*^e^
3 f	35 g	0.98 ± 0.06	0.44 ± 0.03	−0.04 ± 0.05	0.944	132
	28 h	1.43 ± 0.13	0.52 ± 0.03	0.10 ± 0.06	0.954	127
	7 i	0.93 ± 0.12	0.66 ± 0.14	−0.84 ± 0.30	0.974	36
3 j	12 k	1.46 ± 0.19	0.37 ± 0.09	0.10 ± 0.08	0.943	37
	12 l	1.78 ± 0.18	0.43 ± 0.07	0.14 ± 0.06	0.965	61
4	12 m	1.67 ± 0.19	0.31 ± 0.07	0.10 ± 0.09	0.941	35
	5 n	0.80 ± 0.03	0.59± 0.01	−1.31 ± 0.03	0.999	578

a *n* is the number of solvents;

b With associated standard error;

c Accompanied by standard error of the estimate;

d Correlation coefficient;

e *F*-test value;

f Results obtained using the specific rate data from reference [60] at 35.0 °C;

g All available solvents;

h Excluding the data points in the TFE (aq) and HFIP (aq) solutions listed in reference [60];

i Just the TFE (aq) and HFIP (aq) data points in reference [60];

j This work at 25.0 °C.

k This work, excluding the data points in all the TFE (aq) and HFIP (aq) solutions;

l This work, excluding the data points in 80T–20E, 97%–70% TFE (aq) and 97%–70% HFIP (aq) solutions;

m This work, excluding 80T–20E, 70% HFIP, and the three TFE (aq) data points;

n This work, just 80T–20E, 70% HFIP, and 90%–70% TFE.
